# Johann Joseph Oppel (1855) on Geometrical–Optical Illusions: A Translation and Commentary

**DOI:** 10.1177/2041669517712724

**Published:** 2017-06-23

**Authors:** Nicholas J. Wade, Dejan Todorović, David Phillips, Bernd Lingelbach

**Affiliations:** Psychology, University of Dundee, UK; Department of Psychology, University of Belgrade, Serbia; Hemel Hempstead, UK; Lingelbach’s Scheune, Leinroden, Germany

**Keywords:** Oppel, geometrical–optical illusions, orientation, size, area, contrast, bisection

## Abstract

The term *geometrical–optical illusions* was coined by Johann Joseph Oppel (1815–1894) in 1855 in order to distinguish spatial distortions of size and orientation from the broader illusions of the senses. We present a translation of Oppel’s article and a commentary on the material described in it. Oppel did much more than give a name to a class of visual spatial distortions. He examined a variety of figures and phenomena that were precursors of later, named illusions, and attempted to quantify and interpret them.

## Introduction

**Figure 1.**
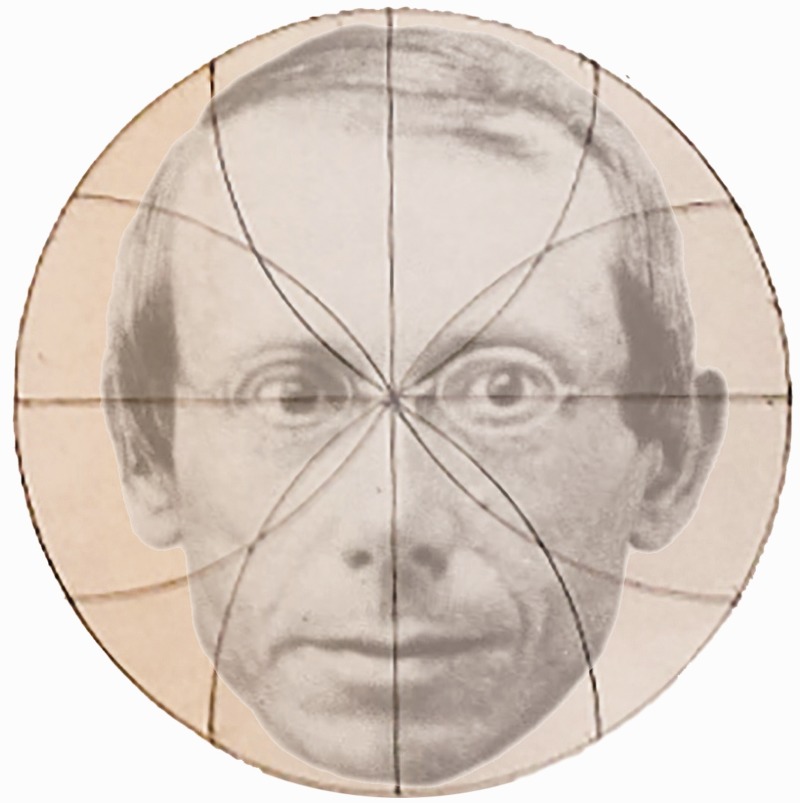
*Oppel’s illusion* by Nicholas Wade. The portrait of Johann Joseph Oppel (1815–1894) is combined with Fig. 12 from Oppel (1855).

Visual illusions have been examined for many centuries and continue to be a source of experimental and theoretical investigations (see Shapiro & Todorović, 2017). Great strides were made from the mid-19th century after Johann Joseph Oppel (1815–1894; Figure 1) defined a subset of spatial illusions that he referred to as geometrical–optical illusions. The contents of the initial article by Oppel (1855) on illusions have been described by several researchers recently (Phillips & Wade, 2014; Todorović, 2017; Vicario, 2011; Westheimer, 2008). As Westheimer (2008) remarked, Oppel’s seminal paper on illusions “appeared in the obscure annual report of the Frankfurt physics club” (p. 2137) and so a translation appears timely.

Oppel (1855) described and displayed various illusions of orientation and size. These included line distortions due to intersecting angles as well as juxtaposed arcs, size distortions as a consequence of line intersections in triangles, rectangles, and circles, as well as the influence of curved figures on size judgments. Two supplementary articles on illusions were published by him (Oppel, 1857, 1861). The first concerned bisection errors, including effects later characterized as the Thiéry-Wundt illusion and the second was addressed to the differences in estimating filled and unfilled extents, now referred to as the Oppel-Kundt illusion. Oppel was an ardent observer of nature and his papers often take their starting point from observations made while walking in the countryside either around Frankfurt or further afield. For example, he observed the motion aftereffect during a trip to the Rheinfall at Schaffhausen (Oppel, 1856); initially he considered that he had discovered the phenomenon, but later deferred to the earlier work on the phenomenon by Plateau, Purkinje, and Müller (Oppel, 1860).

## Life and Work

Brief details of Oppel’s life and a summary survey of his academic work are given in Phillips and Wade (2014). Most of what we can add about his early career chronology is based on a two-paragraph biographical note by Oppel himself in the yearbook of the Frankfurt Gymnasium from autumn 1848; a copy is among the Oppel papers in the Frankfurt town archives (Frankfurt Institut für Stadtgeschichte, Personengeschichte, S2; 2.883).

Johann Joseph Oppel was born in Frankfurt am Main on June 23, 1815. He attended the Weissfrauenschule and the Katharinenschule to be educated as a merchant. His aptitude in mathematics resulted in his transfer to the Gymnasium and he qualified for university entrance in 1833. Between 1834 and 1838, he attended the universities of Giessen and Leipzig where he enroled to study theology and philology. However, he took every opportunity to develop his boyhood enthusiasm for science and mathematics by attending scientific lectures, including some by Moritz Wilhelm Drobisch and Justus von Liebig. Oppel himself later gave popular lectures on astronomy in the Senckenburg Lecture Hall in Frankfurt (Kramer, 1967). He was awarded a Doctorate in Philosophy, Philology, and Mathematics in 1838. Contrary to what was stated in Phillips and Wade (2014), on returning to Frankfurt, Oppel did not immediately return to his old school, the Frankfurt Gymnasium. He taught physics, maths, and Latin at various institutions as well as to private pupils. From 1839 to 1841, he was in Milan as a private tutor for John Frederick Mylius (Spingardi, 2006), a member of the influential Mylius banking and commercial family. From 1842, he resumed private and school tuition in Frankfurt and in 1845, he was appointed an assistant for mathematics and physics instruction in the Gymnasium, becoming a fully established teacher there in 1848 and a full professor in 1859 (Klotzer, 1996). He married Amalie Fritz in 1855 and they had three daughters and a son. Oppel’s sudden retirement in 1878 was a consequence of a severe eye complaint and he died on April 27, 1894.

Despite his significance in the study of illusions, Oppel is not generally known for his science but is celebrated in Frankfurt for his study of the Frankfurt dialect, the *Frankfurter Idiotikon*, which formed the basis for the later, definitive 18 volume *Frankfurter Wörterbuch* (Rauh & Schanze, 1971–1985). He also contributed papers to the Frankfurt literary journal *Diaskalia*. Even the Frankfurt biographical dictionary entry for Oppel (Klotzer, 1996) makes no mention of his scientific work. His papers cover a range of subjects in perception and physics (see Phillips & Wade, 2014).

Oppel was aware of the apparent distortions that accompany many of the simple figures used in teaching geometry. This seems to have been the starting point for his journey into geometrical–optical illusions.

## Geometrical–Optical Illusions

Prior to Oppel’s (1855) article, research on visual illusions tended to be directed to those that occur in the natural environment like the changing apparent size of the moon during its transit, its apparent motion when clouds pass in front of it, the apparent motion of rocks after viewing descending water, and the visibility of afterimages following intense stimulation; spatial illusions tended to be associated with ambiguous figures like those in Roman mosaics (see Wade, 2016, 2017). All this was to change with the move into the laboratory in the early 19th century; this was heralded by the invention of instruments (like the stereoscope and stroboscopic disc) that afforded more stimulus control (Wade & Heller, 1997). Two-dimensional (drawn) stimuli were then enlisted to study stereoscopic vision and motion perception and their application to spatial distortions followed soon after. Before Oppel’s article was published, Fick (1851) described the horizontal–vertical illusion, and this was discussed at some length by Oppel (but without citing Fick). A torrent of slightly different illusions appeared in the remaining decades of the 19th century, many of which bear the names of those who published accounts of them: Zöllner, Poggendorff, Hering, Kundt, Delboeuf, Mach, Helmholtz, Hermann, von Bezold, Müller-Lyer, Lipps, Thiéry, Wundt, Münsterberg, Ebbinghaus, and Titchener. Others were added in the 20th century and yet more variations have been introduced with the aid of computer graphics (see Shapiro & Todorović, 2017).

## Translation

**Figure 2.**
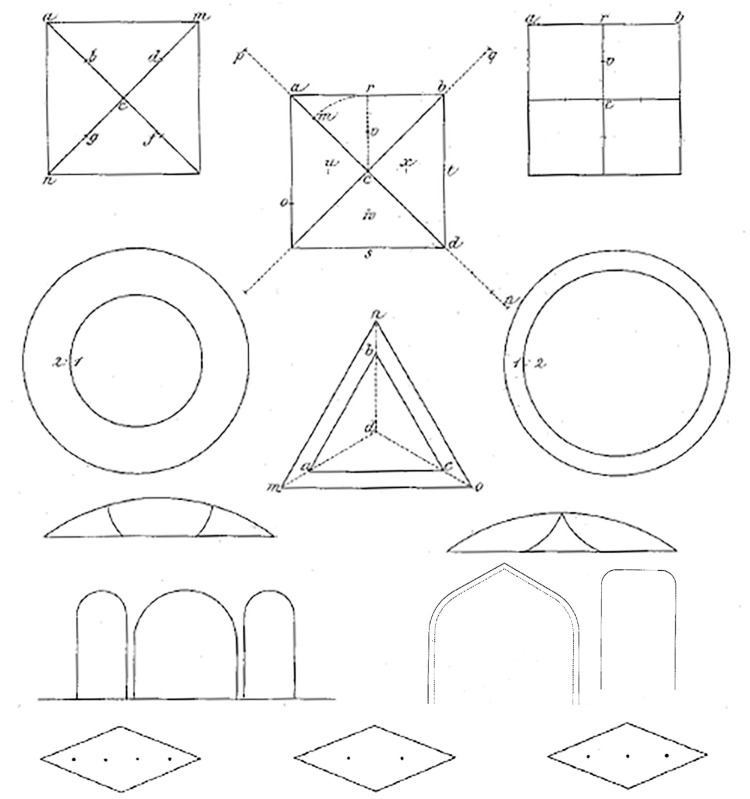
Illusion figures presented in Oppel (1857, 1861). The eight images in the first three rows and the left image in the fourth row are from Oppel (1857); the two images on the right in fourth row and the three images in fifth row are from Oppel (1861).

Oppel is not the easiest of writers to translate, and we have tried to be faithful to his text. It should be noted that the first illustration in his article is numbered Fig. 2; this is because the figures were presented on Plates at the end of the journal together with figures from other articles and they were numbered consecutively. The figures were not accompanied by legends and so we have placed them close to the text in which they are described.

### On Geometrical–Optical Illusions—By Dr. Phil. J. J. Oppel

A quite peculiar class of “optical illusions” relates to those by which the eye—or more precisely, as with all so-called sensory illusions, actually the unconscious judgment of the mind—is in error relative to the dimensions or directions of linear and angular sizes, and therefore they can be appropriately termed geometrical–optical illusions. They are rather sharply differentiated from those that one usually considers to be optical illusions in that for them the reciprocal relations of apparent size (visual angle), actual size, and distance of the seen object have absolutely no relevance, while for the usual so-called *optical illusions*, it is precisely the dependence relations of these three factors that play the main role. If, as is usually the case in the theory of seeing, we call “visual estimation” in the special sense that ability or competence to infer the third one from any two of those three mutually dependent quantities, insofar as the necessary operation of the understanding does not reach consciousness, then an optical illusion (in the ordinary meaning of this expression) is indeed always a misdirection of visual estimation, which, by assuming the value of one of the two given magnitudes as too high or too small, judges the third, the missing one, wrongly. One thinks, for example, of the apparent magnitude of the rising moon compared with it high in the sky; and also the effects in decorative painting.

In the case of the “geometrical–optical” illusions, the distance of the object, and therefore also the angle of vision, as far as it changes with it, has no influence whatsoever, as the following examples illustrate. Several to my knowledge have not been drawn into the field of optical examinations.

To begin with a more familiar case, which is easy to observe, and is often demonstrated by teachers of geometry: vertical dimensions appear to be greater than corresponding horizontal ones. Thus, a right-angled rectangle of 8″ height on a 8½″ base line is readily recognized by the most careful observer as a square, while a real square, placed beside it, appears a little ½″ too high. If students draw by free hand a square on the blackboard, it will appear to the naive observer almost without exception to be too wide (relative to the height). It is just as difficult to make a simple ratio of 1 to 2 in the case of drawing an oblong, and the error is almost always made in the direction of the horizontal dimension (whether it be the larger or the smaller).

The same illusion is manifested in the construction of angles and triangles. An angle of 93° is drawn on a black tablet so that its line of division would run in the vertical direction, and the eye of the naive observer will readily recognize it as a right angle; if the tablet is now turned by a quarter, so that the opening of the angle to the right or to the left, that is, the (imaginary) half-line lies horizontally, the angle immediately appears obtuse. On the other hand, with an angle of about 87°, in the last described position, it is regarded as a right angle, while, after the rotation of the drawing, a quarter-turn is immediately recognized as an acute angle. Conversely, if an isosceles right-angled triangle with a horizontal base is required, the majority of the pupils will draw an obtuse-angled triangle, and if the base is to be vertical, then they will draw an acute triangle.

Likewise, an ellipse which is cut out of white paper and placed on a black ground, the axes of which are 17:18, or even 11:12, is recognized as a circle by naive observers when the longer axes are horizontal, but as soon as the figure is rotated by 90°, it appears elliptical. If, on the other hand, the diameter of a cylinder (such as a glass) standing on a table is to be estimated by eye, such that, for example, the vertical distance between the thumb and the index finger indicates its height above the surface of the table once it is turned (into the horizontal orientation), then when it is actually turned, it turns out that it was judged as too small. The counter-experiment (with the original cylinder and the diameter to be indicated in the horizontal direction) is easy to make.

The fact that even an oblique line is still easily seen as longer than a horizontal one is the result of the difficulty of correctly drawing an equilateral triangle with a horizontal base on a vertical surface: the naïve observer here too makes the base line slightly too long, but the error is recognized immediately when the surface is turned so far that one of its other sides is now horizontal.

The explanation of all these illusions is undoubtedly due to the fact that the eye, by virtue of its normal position with regard to the external world, especially relative to the surface of the earth, is accustomed to see much greater dimensions in the horizontal than in the vertical direction, and because of this slightly overestimates vertical extents compared with horizontal. It is precisely because of this that the apparent exception which the human body sometimes makes from the above rule can be understood; as a person lying flat on the ground appears to most observers longer than when he is in an upright position—(this is because the eye is especially accustomed to see the human figure far more frequently in an upright position, that is, much larger in its vertical than its horizontal dimensions.)—except if here, which also sounds plausible, the visual angle, or rather the angle of incidence of the lines of sight with the principal direction of the body exerts an influence, that is, if in the upright position the perspective foreshortening plays a greater role.^1^

The fact that the latter can indeed give rise to some striking illusions is proved by a well-known experiment with a hat and so forth. (Let a person, unaware of the expected result, be allowed to look at an ordinary man’s cylindrical hat, and then on any wall of the room indicate the height to which the hat, placed on the ground, would appear. The error is, as a rule, so obvious that it excites the astonishment of anyone who has seen it.)

To be sure, in all the experiments mentioned, the results will only be as stated as follows: if the draftsman or observer is really naive, that is, he is not alerted in advance about the direction in which the eye is usually in error since otherwise the conscious effort to prevent the error, will succeed either to avoid it or even to commit the opposite error; this remark, of course, applies to all the following observations.

A second set of these geometrical–optical illusions refers to the relative directions of lines, and in which the horizontal or vertical position does not seem to have an influence. First of all, two observations are worth mentioning, which I owe to the kind communication from Dr. Poppe (Head Teacher at the College of Trade). If a straight line is drawn outside a circle so that it almost touches it, the straight line appears to the naive eye of many observers weakly bent or bowed near the circle, with a convexity toward the circle; the line is thus attracted by the circle, so to speak. Whether the straight line is vertical, or horizontal, or oblique makes no or little difference (inasmuch as the result appears to me to be somewhat more marked when the position is oblique.). If an obtuse angle (at least 135°) (in the manner of Fig. 2) is cut near its apex by a straight line, such that the line would be perpendicular to its (imaginary) bisector, the line appears weakly bent or bowed in the middle, that is, it appears an obtuse angle, the opening of which is opposite to the actual obtuse angle.

**Figure:**
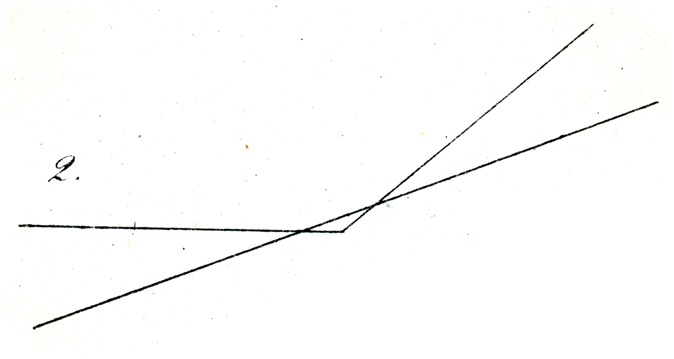

**Figure:**
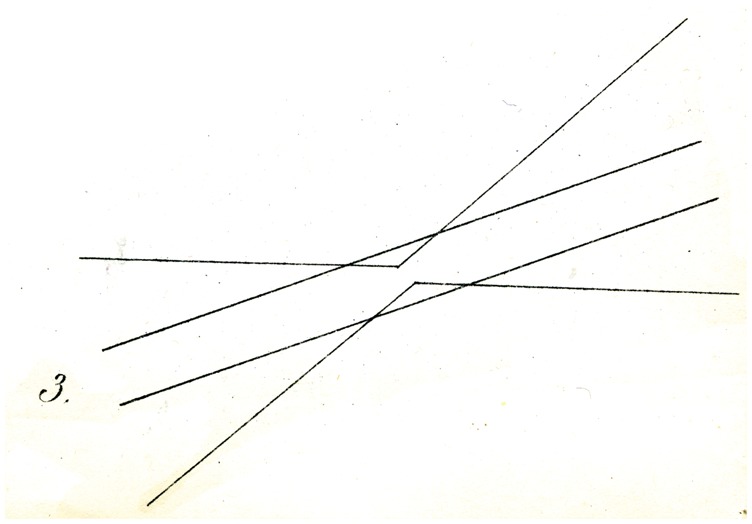

Still more striking is the illusion, when two of the angles described are opposed as in Fig. 3, and they are intersected by two parallel lines: The latter appears distinctly further from each other in their center than on both ends. And here too, the vertical, or horizontal, or oblique position of the figure makes no difference to the occurrence of the described result (though perhaps not quite for its magnitude). It is interesting to note, however, that the counter-experiment shown in Fig. 4, in which the obtuse angle acb (of 135°), is intersected by a different, larger angle mnr (of about 177°), which latter is near to uvw and is equal and parallel to it: then this third angle uvw appears clearly as an angle (i.e., as a bent line at v), but the second (mnr) appears as a straight line.

**Figure:**
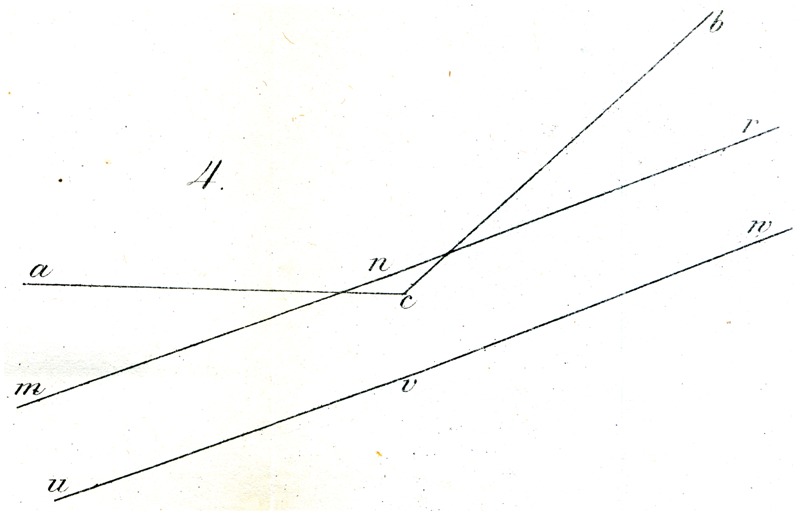

It is not the case, as might be supposed at first sight, that irradiation plays a role in all these observations. I conclude this from the fact that the same results are obtained when the figures are black on white, or white on a black ground. Only, with regard to the size of the angles in question, there seems to be a small degree of variation between the eyes of different observers, since one eye shows a greater, the other a lower sensitivity in the estimation of such directional differences.

Still more striking is the result of the first-mentioned experiment (with the straight line and the circle): If, instead of one, two straight lines and instead of the whole circle only arcs of approximately the same linear length and of about 120° are used, as indicated in Fig. 5.

**Figure:**
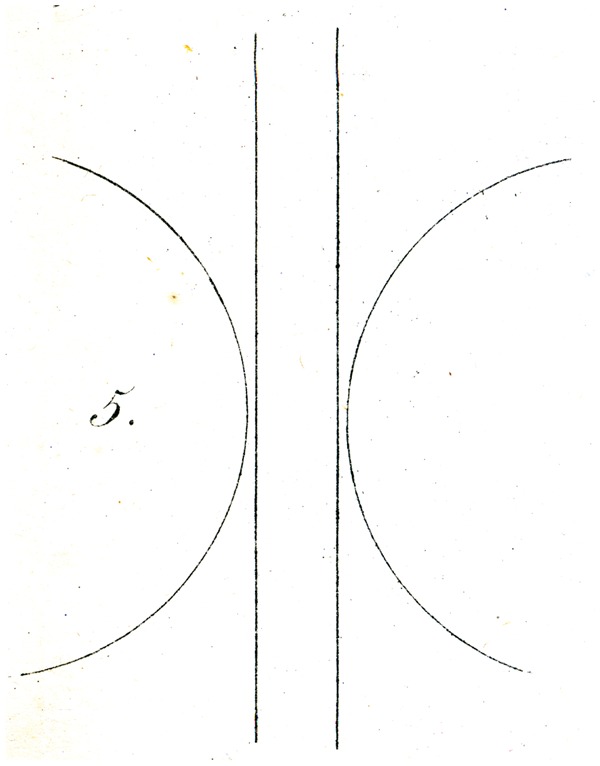

The parallel lines then appear (for any position of the figure) to be distinctly farther apart from each other in the vicinity of the circular arcs than at each end; whereas in the counter-demonstration, Fig. 6, the two middle lines can be fairly strongly bent.

**Figure:**
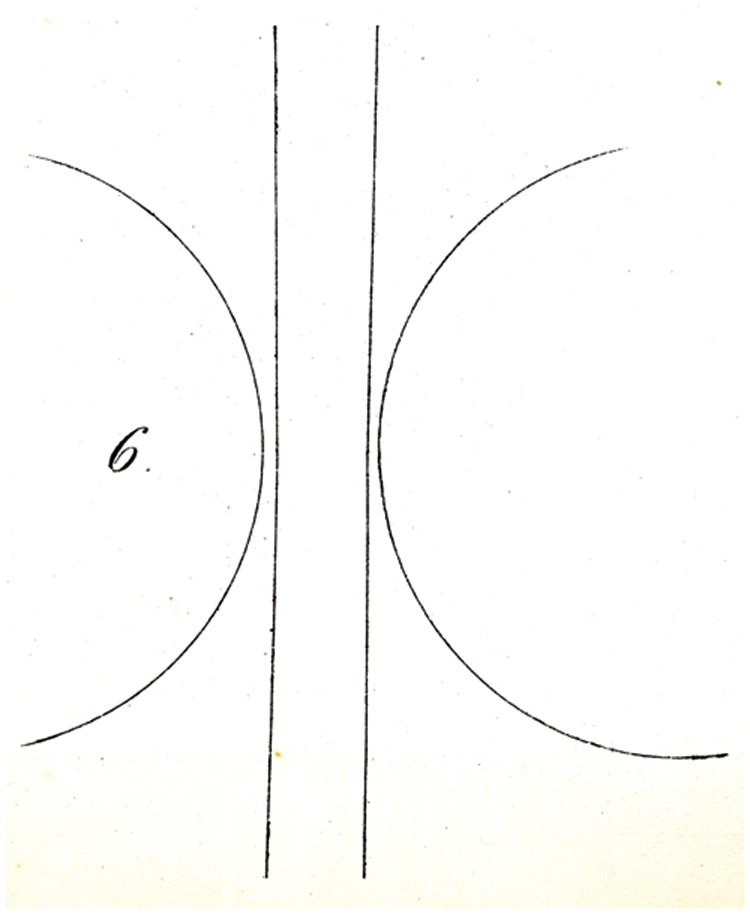

That is, they may be closer together in the middle, without the eye ceasing to maintain them as parallel (both of them form obtuse angles of about 177°). Here too, the results are the same with black lines on white, as with white on black.

Explaining the illusions under discussion is not entirely easy. That physiological processes might be in play is not probable to me. Rather, I might suggest as a proposal, perhaps sufficient, that the eye judges directions (as well as movements) of lines, only through comparisons, and so in general assesses differences in orientation (that is to say angles) as larger wherever judgment of the difference in question is made easier, thanks to closeness of the deviating direction. In this way, for example, in Fig. 4, the difference in orientation between ac and mn (on account of the simultaneous proximity of both lines to the centre of vision) appears greater than the difference between ac and uv, from which comparison, a small (apparent) difference in orientation of mn and uv must result, so that mn is, as it were, moved into alignment with nr*.* Similarly in Figs. 5 and 6, the difference in orientation between the straight lines and the circles in the vicinity of the latter is more apparent to the eye, than at greater distances from them, so that undoubtedly the apparent orientation of both straight lines must be modified (in opposing directions) in the vicinity of the arc. Whether this attempt at explanation is valid or sufficient, I leave to the judgment of the reader. A readily made observation in its support seems to me to be that if one tries to sketch by eye the direction of the streets of a crookedly laid out town, or the intersections at various angles of forest paths (and thus tries to lay out a plan or map or them), the acute angles are as a rule overestimated, and the obtuse angles (insofar as they are apprehended as angles) rather underestimated.

Finally, a third group of optical illusions in this category comprise those, which concern size relationships of simultaneously observed dimensions (irrespective of vertical or horizontal attitude), in particular the division of surfaces and lines.

I submit first the following observation.

Let us stick a strip of black paper about ½″ wide and 10″ to 12″ long on a white surface, and then let a naïve observer lay two quite thin little paper strips of arbitrary length and of the color of the background at right angles over the black strip, and then so adjust their position that the latter appears partitioned into three equal segments. If now we check the accuracy of this partitioning with compasses, we will as a rule observe, that—at least at a first attempt—the two outer segments, in comparison with the middle one, will turn out rather too large. The same happens with a white strip of paper on a black ground—evidence, therefore, that in this case irradiation (in the usual sense of the word) plays no role. In the same way, and at the same time still more strikingly, we obtain the same result, if the strip is divided not into three, but (with three transverse strips) into four apparently equal parts, or alternatively is left partitioned into three, of which the middle one is double in extent.

The result of this test is, no doubt, essentially in agreement with the extent to which visual estimation of an observer (in the broad sense of the term) is sharpened by practice, as well as by the amount of time and care taken in the equal partitioning of the adjacent segments. The result in question is still more clearly and certainly apparent, when we do not have the observer carry out the partitioning himself, but set before him previously partitioned strips for the assessment. Should we choose, for example, two strips, of which one has been divided exactly into three equal parts (with fine transverse lines) and the other so divided that the middle segment is just a very little too short, the majority of observers will judge the latter as the one equally partitioned; whereas to the contrary no error occurs, when beside the correctly partitioned strip we place a second, in which the middle segment is a little too long. Most convincingly of all, finally, is a test with two such strips, one of them with the middle segment a little too large and the other by an equal amount too small: in this case, all observers judge the latter as more accurately partitioned.

In a similar way, if we divide for example an octavo sheet of paper with vertical lines into three columns of equal width, the two outer ones appear somewhat smaller than the one in the middle.

To convince myself of the extent to which the thickness of the partitioning lines might have some influence, I repeated the tests described with strips which were partitioned (not with very fine lines, but) with black lines of 0.1″ width, taking care however to add to both ends of the partitioned paper strip half the width of such partitioning lines (0.05″) also in black color, so that the intervening spaces left white were equal- or were unequal, in the way described: the result was however exactly the same (as also against a grayish or brownish background).

When by chance I mentioned these observations to a skilled and experienced artist, they did not really come to him as news: rather, he confirmed my belief that we can note similar visual misjudgments with all equally partitioned surfaces (the facades of a building, colonnades, etc.) in that consistently the outer-lying parts, adjacent to the “air,” appear somewhat smaller, because as he put it in his over-blown artistic terms, “each time the air gobbles them up,” that is to say, the background decreases the apparent extent of the surfaces lying adjacent to it; and indeed that allowance was made already in ancient architecture, consciously or instinctively, for this tendency of the eye. Irradiation does not appear to me responsible in this case on the grounds already set forth earlier: rather, I am much more inclined here again to invoke an effect on the extended surface of the background which through greater or lesser proximity facilitates or inhibits comparison.

A second experiment that is relevant here is the following:

Suppose we construct with fine white, or alternatively black strips, on an accordingly black or white background, two obtuse triangles with unequal sides of, for example, 7″, 12½″, and 16″, so that the latter edge forms the base of the triangles. In one of these two triangles, we divide this base exactly and draw up from the midpoint a transversal (of the same color as the sides of the triangles) to the apex. In the other by contrast, we draw the transversal from a point on the base, which lies about 1/4″ nearer to the smallest angle. If we now ask a naive observer, before whom we lay out the two triangles side by side, in which one the bisection of the base appears accurate, he will as a rule choose the latter, in which one half is longer than the other by 1/2″ (see Figs. 7 and 8).

**Figure:**
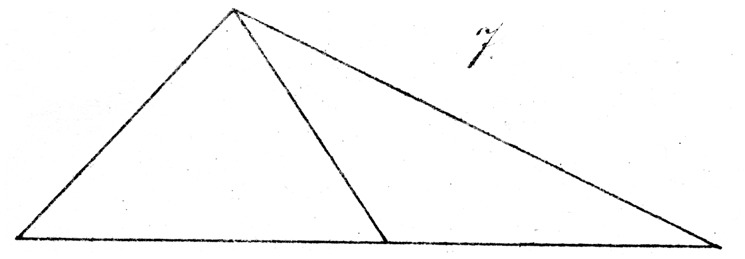

**Figure:**
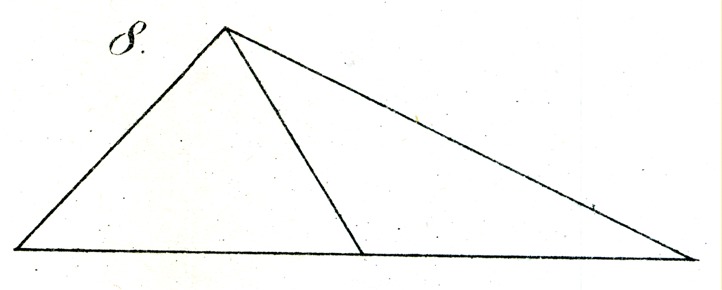

Some small variations on the experiment, with moveable transversals, will suggest themselves easily. However, this will generally only succeed if we involve only such persons who do not through practice possess truly experienced visual estimation, and even in that case the correct relationship will often be reported as a consequence of somewhat longer and more careful observation.

On the other hand, the illusion remains robust in the following case. We construct in the same way two congruent obtuse triangles (as in Figs. 9 and 10) with sides, for example, of 7″, 15″, and 10″ (once again with the last as the horizontal base) and divide the base as before with a transversal, in one triangle exactly, in the other shifted about 0.4″ from the middle toward the obtuse angle.

**Figure:**
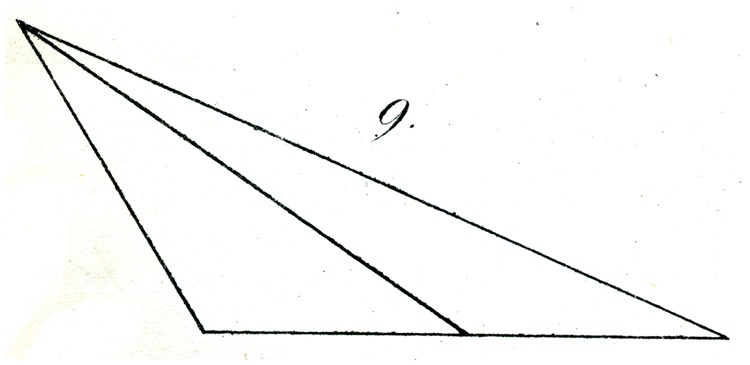

**Figure:**
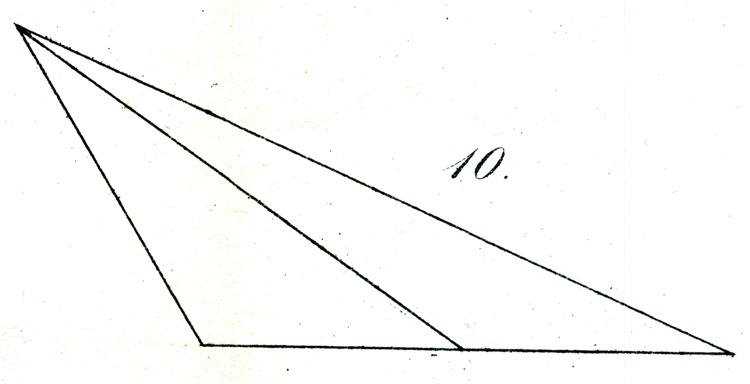

Here again, the question as to which base is most correctly bisected is as a rule answered in error. The illusion arises in this case, in comparison with the previous experiment, exactly in the opposite sense; yet, the explanation confirms that already advanced. That is to say, the eye apparently misassociates the bisection of the (small) angle at the apex with the bisection of the base, and holds the bisection of the latter in that case for more accurate, in which the partition of the former approaches the correct bisection.

Still more striking is finally the following observation, which I have regularly made when teaching geometry.

We draw in a circle of arbitrary radius (as in Fig. 11), two diameters at right angles, and from the ends of each construct arcs, of the same radius, to the extent that they fall within the aforementioned circle, whose circumference is, as is well known, divided into 12 equal arcs (of 30°).

**Figure:**
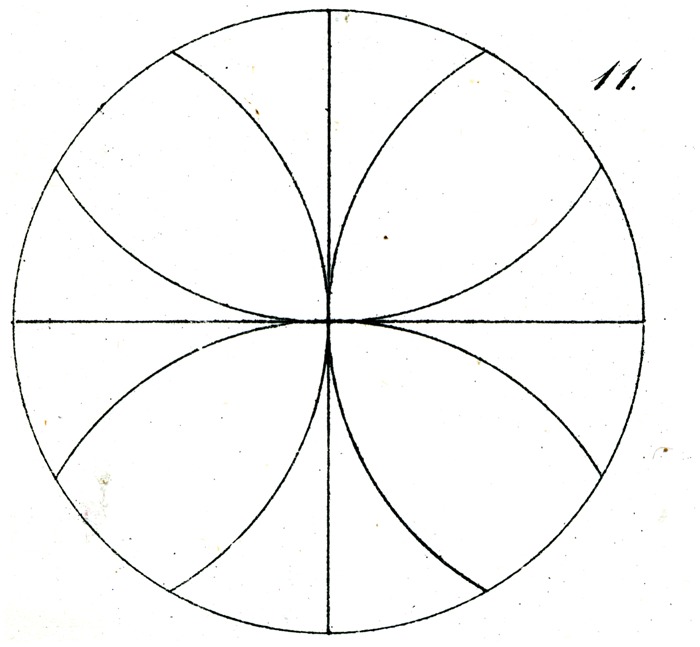

The equality of these 12 segments appears, however, to most pupils at first glance so unbelievable, with, in particular, the middle of the three segments in each quadrant appearing so much larger that they are disinclined to try to prove the correctness of the geometrical construction.

The basis for the illusion here again lies in the partial misassociation by the eye of the partitioning of the area of the quadrant with that of its perimeter, misled by the inequality of the former to judge the latter also as unequal. We see this immediately if, having made the 12-fold division of the circumference, the inner arcs (of the same radius) are inverted that is to say drawn out as from points m, n, r, s, and so forth as in Fig. 12 (now naturally as arcs of only 60°) whereby the illusion immediately vanishes or is even reversed.

**Figure:**
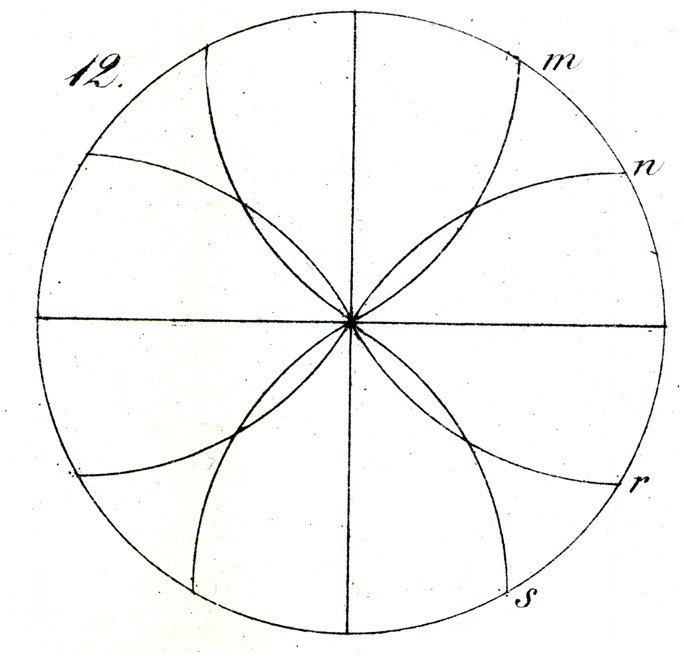

The correctness of the explanatory principle indicated follows also from a related simplification of the experiment, which, for example, might take the form that we bisect the two longer sides of a rectangle with an arc, as shown in Fig. 13, whose center lies in the middle of one of the two smaller sides: here again, the eye readily attributes to the division of the contours the inequality of the partitioning of the area.

**Figure:**
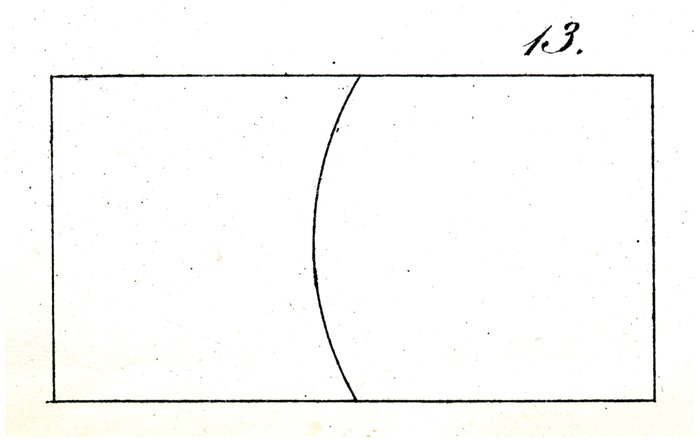

A similar illusion appears if, as shown in Figs. 14 and 15, parallel lines are divided into three segments with arcs, as will be apparent with just a glance at the figures, and so forth.

**Figure:**
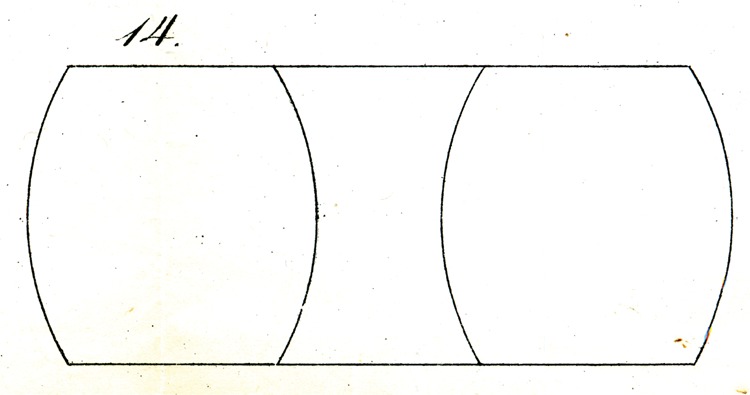

**Figure:**
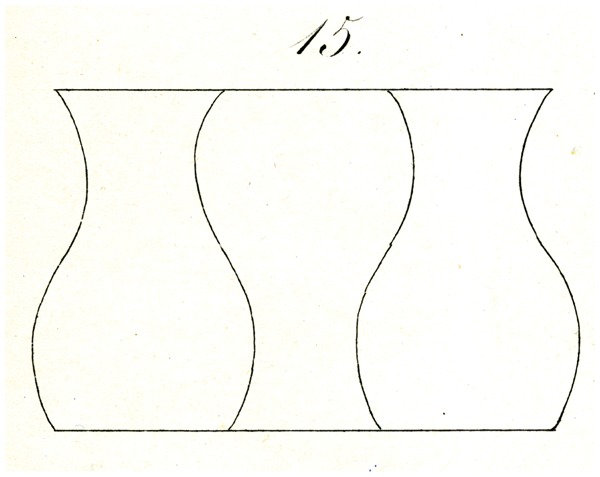

That the same principle also applies with partitioning using straight lines under similar circumstances is shown, for example, in Fig. 16, where a perpendicular constructed from the middle of the base of an obtuse triangle appears strikingly nearer to the apex of the acute angle and so forth.

**Figure:**
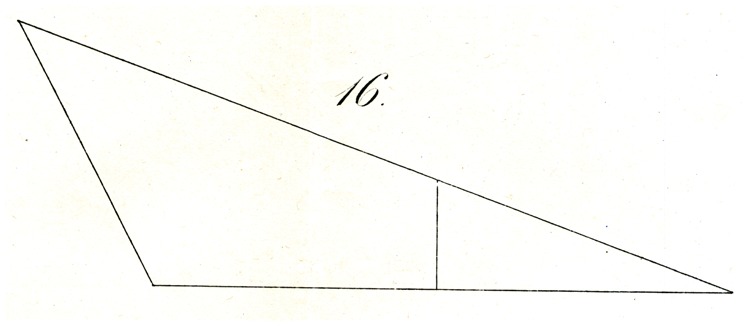

## Commentary

Oppel gave the simple figures that induced spatial distortions the label geometrical–optical illusions in order to confine them to the relatively small but reliable distortions of visual space, mostly in the domains of size or orientation. This implicit classification has proved too restrictive as some combine both dimensions and others involve different dimensions altogether. Vicario (2011) listed 26 different classifications! Perhaps the simplest is the one proposed by Boring (1942): extent, direction, and the rest. The common factors of the classifications are direction (orientation), size, contrast, assimilation, and perspective. Some systems are concerned with the distorted dimensions (like size and orientation), others involve possible underlying processes (like assimilation and contrast, and eye movements), and yet others characterize the levels at which the illusions should be considered (like physical, physiological, and psychological). Oppel touches on all these factors in his article.

In his introductory remarks, Oppel states that geometrical–optical illusions are clearly different from what are usually called optical illusions, such as in the moon illusion. He considered that the other optical illusions are characterized by the relations of three quantities, apparent size (visual angle), real size, and distance of objects, and they are based on the fact that if one of a pair of these magnitudes is wrongly set too high or too low, the third one is judged incorrectly. In contrast, geometrical–optical illusions are not influenced by either the distance or the visual angle of the display.

His account commences with the observation that vertical dimensions generally appear larger than corresponding equal horizontal dimensions. The discussion gives the impression that the phenomenon was in common currency among teachers of geometry so that there was no need to cite Fick (1851). Indeed, Oppel’s article is free from references to others. His examples of horizontal–vertical illusions include a variety of figures like rectangles, ellipses, cylinders, and triangles. Oppel claimed that the explanation of all these phenomena is “without doubt” the fact that the eye is accustomed to see much larger extents in the horizontal than in the vertical orientation, and thus out of habit slightly overestimates the vertical extents. This leads him to examine figures which display apparent angle expansion, contour attraction, and repulsion as well as illusions of extent due to line intersections.

Some of Oppel’s designs (Figs. 11 to 15) bear a certain similarity to the famous configuration introduced much later by Müller-Lyer (1889). This relation was noted by Wundt (1898) and Todorović (2017). Müller-Lyer himself was apparently unaware of Oppel’s work, since he did not cite Oppel in his papers. There are similar precursors to the orientation illusions associated with Zöllner (1860). One of Oppel’s colleagues noted that when two lines forming an obtuse angle cross a straight line, the latter looks as if slightly bent or broken (his Fig. 3). A similar small bulging effect appears when the two parallel lines are almost touched by circular arcs (Fig. 5).

Oppel followed his ground-breaking paper of 1855 with two more on geometrical–optical illusions (Oppel, 1857, 1861) illustrations from which are shown in Figure 2. Nine figures were included in the first supplement (eight images in the first three rows, and left image in the fourth row in Figure 2), and several types of illusion were discussed. One involved errors in locating the midpoints of lines embedded in simple geometrical configurations (left and middle image in the first row). This effect is closely related, if not identical, to the Thiéry-Wundt illusion (Anstis, Gregory, & Heard, 2009; Day & Kim, 2010; Thiéry, 1896; Wundt, 1898) and Judd’s illusion (Judd, 1899; Morgan, 1969). For some examples and variations of this effect, see Todorović (2014, 2017). Oppel not only presented graphical illustrations for the readers to inspect but also reported quantitative data in the form of the number of observers who experienced the illusion (in one case 85 out of 88). In addition, he included a control experiment with a related configuration for which there was no illusion (right image in first row). Another type of effect involved errors in the judgments of areas of geometrical figures (three images in second row). One of his several examples was a circle surrounded by an annulus which has the same area as the circle; his students stated that the area of the annulus looked smaller than that of the circle. He also claimed that if a sequence of figures is presented all of which have approximately the same area (a triangle, a square, a pentagon, a hexagon, a heptagon, an octagon, and a circle), then the members of the sequence look increasingly smaller in area (both to naïve and trained observers, but to the latter only when they inspected the figures casually). He also reported that some observations on perceived lengths of segments of lines (illustrated in two images in the third row), which, like some figures in the first paper, were anticipations of the Müller-Lyer illusion.

The second supplement (Oppel, 1861) included five illustrations (the two images on the right in row 4 and the three images in row 5 in Figure 2) and was also concerned with a number of phenomena. He was surprised when he saw a professional painter use a compass to measure the correct distance to paint the reflection of a rock from the water level. The painter explained that the reason was to avoid an illusion, whose presence Oppel confirmed for himself when his judgment of the distance turned out to be too short. He also claimed that in several wall frescos from Pompeii depicting Narcissus admiring his own reflection in a pool, the reflection was painted too high. Prompted by these observations, Oppel conducted a study with 40 observers who each made 10 quantitative judgments on various configurations, which he described but did not illustrate, and whose data were reported in full in a table. However, the results turned out to be somewhat ambiguous. Another effect involved distributing several equidistant dots along the longer diagonal of a rhombus, and noting that the distances nearest to the corners looked shortest (the three images in the bottom row). This observation anticipates the results of Warren and Bashford (1977), Post, Welch, and Caufield (1998), and Predebon (2001), all of whom found that the illusory shortening of the shaft in the wings-in version of the Müller-Lyer illusion is not uniform but confined to the region near the vertices of the wings. Finally, Oppel devoted less than a page, without any accompanying figures, to the observation that undivided lines or areas appear smaller than when they are divided, an effect now known as the Oppel-Kundt or filled-space illusion (Kundt, 1863; Wackermann, 2017). It is ironic that the only geometrical–optical illusion with which Oppel’s name is linked is one that he did not illustrate. His example involved squares which, as he already noted in the 1855 paper, are drawn wider (horizontally) than they are tall (vertically). Here, he noted that this difference is even larger when the task is to draw a square on a paper with horizontal lines (with the side of the square being equal to four or five times the distance between the lines), so that the square is divided into several parts in the vertical direction. Oppel realized that in this case, the effect is confounded with the general exaggeration of the vertical dimension, described in his first paper, and suggested an “easy control experiment” in which the lined paper is turned by 90°, so that the lines are now vertical. He noted that in this case, the width of the drawn figure is still wider than its height, but to a smaller extent than before. In both papers, Oppel reported some observations on architectural details, illustrated in the fourth row of Figure 2.

## Conclusion

Oppel (1855) performed a service to visual science by defining a subset of illusion figures that have been investigated intensively ever since. In addition, he presented precursors of orientation and size illusions that are associated with the names of later researchers.

## Note

1 If half the body length is taken to be a unit of measure, and if the distance of the eye of the observer from the middle part of the length, expressed in such units, = e, then, as a simple trigonometrical derivation shows, the relation of the tangent of the visual angle of the lying body (viewed from the side) to the tangent of the visual angle for the upright body, is as e: √(e^2^−1), that is, quite exactly as 2e^2^: 2e^2^−1; the latter is thus always smaller.
